# Cognitive and Biomarker Signatures of Late-Onset Temporal Lobe Epilepsy: Toward Non-Alzheimer Neurodegenerative Mechanisms

**DOI:** 10.1212/WNL.0000000000218356

**Published:** 2026-08-04

**Authors:** Alessia Casarini, Alice Ballerini, Riccardo Maramotti, Manuela Tondelli, Chiara Carbone, Annalisa Chiari, Giulia Vinceti, Roberta Bedin, Teresa Urbano, Marcella Malagoli, Maurilio Genovese, Simona Scolastico, Giada Giovannini, Matteo Pugnaghi, Niccolò Orlandi, Maria Angela Molinari, Stefano Meletti, Giovanna Zamboni, Anna-Elisabetta Vaudano

**Affiliations:** 1 Department of Biomedical, Metabolic and Neural Science, University of Modena and Reggio Emilia, Italy;; 2 Neurology Unit, OCB Hospital, Neuroscience Department, Azienda Ospedaliera-Universitaria, Modena, Italy;; 3 Neuroradiology Unit, OCB Hospital, AOU Modena, Italy;; 4 Diagnostic Imaging, Villa Rosa, Modena, Italy; and; 5 Neurophysiology Unit and Epilepsy Center, Neuroscience Department, Azienda Ospedaliera-Universitaria, Modena, Italy.

## Abstract

**Background and Objectives:**

Late-onset unexplained epilepsy (LOUE) represents a substantial proportion of epilepsies with onset after 50 years and often manifests as temporal lobe epilepsy (LO-TLE). Although a link with Alzheimer disease (AD) has been suggested, only a subset of LO-TLE shows AD-related biomarkers, indicating biological heterogeneity. This study aims to characterize the cognitive and CSF phenotype of LO-TLE and compare it with healthy controls (HCs) and patients with mild cognitive impairment due to AD (MCI-AD).

**Methods:**

This Italian cross-sectional cohort study included LO-TLE patients with normal CSF β-amyloid (Aβ) biomarkers, MCI-AD, and age-matched and sex-matched HC. Participants underwent structural MRI, neuropsychological assessment, and CSF biomarkers assay, including neurofilament light chain (NfL) and the phosphorylated-to-total tau ratio (p/t-tau). Cortical thickness and subcortical volumes were quantified from structural MRI. Cognitive performance was summarized using principal component analyses. Group differences in imaging, cognition, and CSF biomarkers were assessed, and associations between CSF markers and cognition were examined within groups.

**Results:**

The study included 18 LO-TLE, 24 MCI-AD, and 17 HC. LO-TLE showed preserved cortical thickness and subcortical volumes comparable with HC, whereas MCI-AD exhibited widespread cortical thinning and medial temporal atrophy. Despite normal imaging, LO-TLE showed lower performance compared with HC in episodic memory (*t*_(53)_ = −7.79, *p*_FDR_ < 0.001), short-term memory (*t*_(53)_ = −2.94, *p*_FDR_ = 0.007), language (*t*_(53)_ = 4.12, *p*_FDR_ < 0.001), and executive functions (*t*_(53)_ = −3.76, *p*_FDR_ < 0.001), while attention was preserved. Global cognitive performance further distinguished LO-TLE from MCI-AD, with the former group performing better (*t*_(53)_ = 4.21, *p*_FDR_ < 0.001). LO-TLE CSF profiles were characterized by low NfL levels and a p/t-tau ratio below the proposed cutoff of 0.17, whereas MCI-AD showed pathologic Aβ and tau alterations, elevated NfL, and p/t-tau ratio above 0.17. In LO-TLE, a higher p/t-tau ratio was associated with better performance on global cognition (*rs* = 0.585, *p*_FDR_ = 0.032) and short-term memory (*rs* = 0.588, *p*_FDR_ = 0.032), whereas no associations emerged in MCI-AD.

**Discussion:**

LO-TLE with normal CSF AD biomarkers is characterized by distinct cognitive and biological features compared with MCI-AD, suggesting a disease process independent of AD. The low p/t-tau ratio may reflect alternative pathophysiologic mechanisms and warrants further investigation in larger longitudinal studies to clarify the underlying pathology and clinical trajectories.

## Introduction

Epilepsy is a common neurologic disorder displaying a characteristic U-shaped distribution with incidence peaks in early childhood and in the elderly.^1^ In older adults, the leading causes of epilepsy are mainly cerebrovascular disease and brain tumors; however, a considerable proportion of cases, ranging from 33% to 53%, occur without an identifiable etiology.^2^ These cases are collectively referred to as late-onset unexplained epilepsy (LOUE), among which temporal lobe epilepsy (LO-TLE) represents one of the most frequent clinical subtypes.^3^ LOUE and Alzheimer disease (AD) are increasingly recognized as interrelated conditions sharing common pathophysiologic mechanisms.^4^ Patients with mild cognitive impairment (MCI) or AD have an increased risk of seizures, with lifetime prevalence ranging from 20% to 64%.^5,6^ Multiple studies report that individuals with LOUE have a higher risk of developing MCI or dementia than age-matched individuals without epilepsy.^7^ Reduced CSF β-amyloid (Aβ) levels and increased phosphorylated tau (p-tau) concentrations have been reported in LOUE, with up to one-third of patients displaying an AD-like CSF biomarker profile.^4^ These alterations suggested the hypothesis that in a subset of patients, LOUE may represent an early manifestation of AD-related neurodegeneration. However, LOUE is increasingly recognized as a clinically and biologically heterogeneous condition rather than a single-disease entity. Evidence indicates that LOUE includes forms with and without neurodegenerative biomarker abnormalities.^8^ This heterogeneity mirrors the clinical variability in LOUE^9^ and highlights the need for more refined phenotypic characterization. In our previous work, we showed that LO-TLE patients with normal CSF AD biomarkers display preserved cortical and subcortical morphology, clearly distinguishing them from MCI due to AD (MCI-AD).^10^ These findings suggest that LO-TLE itself may not be an intrinsic risk factor for AD-related neurodegeneration and underscore the importance of integrating clinical phenotyping, brain morphometry, and CSF neurodegenerative biomarkers to accurately characterize this population. Building on these results and the same cohort, this study substantially extends our previous work^10^ by providing a comprehensive neuropsychological characterization and by expanding the CSF biomarker assessment with neurofilament light chain (NfL) and the phosphorylated-to-total tau (p/t-tau) ratio, biomarkers not previously investigated in the LO-TLE population. Accordingly, we addressed the following questions: (1) do LOUE patients with normal CSF AD-related biomarkers show cognitive impairment compared with healthy controls (HCs)? (2) Does their cognitive profile differ from patients with MCI-AD? (3) Can their biological profiles be further refined through the consideration of these additional CSF biomarkers? We hypothesized that patients with LO-TLE present cognitive impairments despite normal AD-related CSF biomarkers and that their biomarker profile may point toward a neurobiological mechanism independent of the AD process.

## Methods

### Participants

This observational study included 2 groups of patients, LO-TLE and MCI-AD, retrospectively selected from a previously published cohort.^10^ Patients with LO-TLE were evaluated at the Epilepsy Center at the Modena Academic Hospital (Azienda Ospedaliero-Universitaria di Modena, Italy). Patients with MCI-AD were recruited through the Cognitive Neurology Clinic at the same institution. In both cases, patients were referred by general practitioners, neurologists, or other specialists within the regional health care network for diagnostic evaluation and management. For patients with LO-TLE, inclusion criteria were (1) epilepsy onset after 50 years, consistent with epidemiologic data^11^; (2) diagnosis of TLE according to the International League Against Epilepsy (ILAE) criteria^12^ confirmed by expert epileptologists (S.S., G.G., M.P., N.O., S.M., and A.E.V.); (3) evidence of unilateral or bilateral temporal epileptiform activity (ictal and/or interictal) on long-term video-EEG monitoring; (4) availability of high-resolution 3D T1-weighted (3D-T1) and 3-dimensional fluid-attenuated inversion recovery (3D-FLAIR) sequences; and (5) normal CSF AD biomarkers, according to our laboratory-specific established cutoffs^13,14^: Aβ_1–42_ >600 pg/mL, Aβ_1–42/1–40_ ratio >0.069, total tau (t-tau) <400 pg/mL, and phosphorylated tau 181 (p-tau_181_) <56.5 pg/mL. Patients with a history of other significant neurologic or psychiatric disorders, a preexisting diagnosis of dementia, use of acetylcholinesterase inhibitors, or a Mini-Mental State Examination (MMSE) raw score lower than 26^15^ were excluded. 3D-FLAIR images were inspected for white matter disease load (i.e., Fazekas score)^16^ by 2 expert neuroradiologists (M.M. and M.G.), with scores defined as follows: (0) none or a single lesion, (1) multiple punctate lesions, (2) beginning confluence, and (3) large confluent lesions.

Inclusion criteria for the MCI-AD group were (1) diagnosis according to Petersen criteria^17^; (2) AD-specific CSF biomarker alterations, confirmed by expert cognitive neurologists and neuropsychologists (A.C., M.M., C.C., M.T., G.V., and G.Z.); (3) availability of a 3D-T1; and (4) absence of any history of seizures and/or epilepsy. Patients with dementia or other neurologic conditions that could impair cognition (e.g., hydrocephalus and multiple sclerosis) were excluded.

A common inclusion criterion across both patient cohorts was the availability of a comprehensive neuropsychological assessment within 6 months of lumbar puncture. To address minor inconsistencies between LO-TLE and MCI-AD cognitive testing, a group of age-matched and sex-matched HCs was prospectively recruited between November 2024 and December 2025 from the same geographical area as the patient cohorts. HC participants underwent a full neuropsychological assessment, ensuring complete and comparable cognitive domain coverage across groups. HCs were included based on the following criteria: (1) no history of neurologic disease or subjective cognitive complaints, (2) availability of a 3D T1-weighted MRI, and (3) no use of psychotropic medications at the time of MRI.

### Image Acquisition and Analysis

3D-T1 sequences were acquired on a 3T GE Healthcare MRI scanner according to the HARNESS protocol.^18^ Cortical and subcortical features were estimated using FreeSurfer (v7.3.2). We extracted vertexwise cortical thickness from template-aligned cortical surfaces and 16 subcortical volumes. Hippocampal subfields and amygdalae subnuclei were segmented using dedicated FreeSurfer pipelines^19,20^ and previous studies from our group^10,21-23^ (eMethods).

### Neuropsychological Assessment

The neuropsychological evaluation assessed 5 cognitive domains: episodic memory, short-term memory, language, attention, and executive functions. *Episodic memory* was evaluated using the Free and Cued Selective Reminding Test (FCSRT)^24^ from which the Immediate Total Recall (ITR) and Index of Sensitivity to Cueing (ISC) scores were derived, measuring spontaneous and cued recall, respectively. Additional tests included the Short-Story Recall Test^25^ to assess verbal episodic retention and the delayed recall of the Rey-Osterrieth Complex Figure (ROCF)^26^ for nonverbal visual memory. *Short-term memory* was measured using the Digit Span Forward and the Corsi Block-Tapping Test (Corsi Test),^27^ assessing verbal and visuospatial spans, respectively. *Language* abilities were examined with the Italian version of the Boston Naming Test^28^ for naming, and Phonemic and Semantic Fluencies^29^ for lexical access. *Executive functions* were explored using Raven Colored Progressive Matrices^30^ for nonverbal reasoning, the Trail Making Test Form B^31^ for cognitive flexibility, and the Frontal Assessment Battery^32^ for higher-order executive control. *Attention* was evaluated through the Stroop Test^33^ for selective attention, the Trail Making Test Form A,^31^ and the Attentional Matrices^34^ for visual scanning and processing speed. Three additional measures were included: the ROCF copy^26^ to assess visuoconstructive and praxis abilities, the Digit Span Backward^27^ as an index of working memory, and the FCSRT Immediate Free Recall (IFR)^24^ reflecting initial verbal learning. These tests were not included in the main cognitive domains but were analyzed in the single-test comparisons.

### CSF Biomarkers

CSF samples were available for LO-TLE and MCI-AD only. Standard International Procedures for CSF biobanking were strictly followed^35^ as previously detailed.^36^ Biomarker concentrations were measured using the CLEIA method (Lumipulse G600II), according to the manufacturer's instructions (Fujirebio Inc., Ghent, Belgium), by experienced biologists (R.B. and T.U.) specialized in AD diagnostics. From the CSF samples, the following biomarkers were analyzed: Aβ_1–42_, Aβ_1–42/1–40_ ratio, t-tau, and p-tau_181_. The CSF p/t-tau ratio was calculatedand the proposed cutoff value of <0.17 was adopted. ^37^ CSF NfL levels were measured using Lumipulse G NfL chemiluminescent enzyme. Refer to the eMethods for more details. In epileptic patients, the lumbar puncture was conducted at least 24 hours apart from the latest recorded or referred seizure, with a range of 24–120 hours.

### Statistical Analysis

#### Demographic and Clinical Variables

Group differences in demographic and clinical variables were analyzed using SPSS (IBM, Chicago, IL), with a significance threshold of *p* < 0.05. Analyses of variance were used for age and years of education, and a χ^2^ test for sex distribution. Independent-sample *t* tests compared age at onset, and the Mann-Whitney *U* test was used for disease duration between patient groups. MMSE scores were analyzed using analysis of covariance (ANCOVA), including age, sex, and education as covariates.

#### Cortical Thickness and Subcortical Volumetry Analysis

Cortical thickness and subcortical volumes were compared across all groups, and all brain measures were *z*-scored based on the HC's mean and SD. To assess seizure lateralization, data from patients with right-lateralized LO-TLE were flipped to align all morphometric measures to the left hemisphere.^21,22^ Thus, all analyses and results are presented as ipsilateral or contralateral to the LO-TLE epileptogenic focus. This approach was not applied to the MCI-AD due to the lack of a specific lateralizing pattern in neurodegenerative diseases. Vertexwise cortical thickness maps were analyzed with BrainStat^38^ on MATLAB (R2021b) with age, sex, and education included as covariates. Subcortical volumes were analyzed with multivariate ANCOVA using SPSS software, with age, sex, education, and intracranial volume included as covariates. Statistical significance for all comparisons was set at *p* < 0.05, with *p* values adjusted using a 5% false discovery rate (FDR).^39^

#### Cognitive Domains Analysis

Missing neuropsychological data were imputed using predictive mean matching within a multiple imputation model. Time-based measures were transformed using the negative logarithm, and all continuous cognitive variables were standardized as *z*-scores relative to HC; negative values reflect cognitive impairment. A principal component analysis (PCA) was performed on the standardized scores; the first principal component (PC1) was retained as a composite measure of global cognitive performance (hereafter “global cognition”), and the procedure was repeated separately for each of the 5 cognitive domains. Group differences were assessed using linear models with pathology as the main factor and age, sex, and education as covariates. Significant effects were followed by pairwise comparisons of estimated marginal means, with FDR correction for multiple testing.^39^ Full methodologic details are provided in the eMethods.

#### Cognitive Performance on Individual Tests

Two separate analyses were performed on individual neuropsychological tests. First, scores were converted to *z*-scores using HCs as the normative reference and compared between groups. Second, binary outcomes were assessed based on Italian normative cutoffs by calculating the proportion of impaired performance. Further details are provided in the eMethods. Finally, patients were categorized into MCI subtypes^9^ as amnestic single-domain MCI (a-sd MCI), nonamnestic single-domain MCI (na-sd MCI), amnestic multiple-domain MCI (a-md MCI), or nonamnestic multiple-domain MCI (na-md MCI).^17^

#### CSF Biomarkers and Correlations

Normality was assessed using the Shapiro-Wilk test. Given the non-normal distribution of the data, CSF biomarker group differences between LO-TLE and MCI-AD were assessed using nonparametric ANCOVA with age as a covariate through the “npANCOVA” package in R. Specifically, t-tau, p-tau_181_, p/t-tau ratio, Aβ_1–42_, Aβ_1–42/1–40_ ratio, and NfL concentrations were analyzed. Moreover, according to the A/T framework,^40^ amyloid (A) and tau (T) status were defined using the Aβ_1–42_, Aβ_1–42/1–40_ ratio, and p-tau_181_, respectively. Finally, we explored possible associations among biomarkers, cognition, and between biomarkers and cognitive measures. Spearman correlations were computed separately for LO-TLE and MCI-AD for the biomarker-biomarker, cognition-cognition, and biomarker-cognition associations using pairwise complete observations. To account for multiple comparisons, *p*-values were adjusted using FDR correction.^39^

### Standard Protocol Approvals, Registrations, and Patient Consents

The study was approved by the Local Ethics Committee of Area Vasta Emilia Nord (N. 155/14 and N. 238/23 for patients with LO-TLE; N. 832/18 for patients with MCI; N. 134/14 and N. 679/22 for healthy participants). Written informed consent was obtained from all participants. The study was conducted in accordance with the principles outlined in the Declaration of Helsinki.

### Data Availability

The data that support the findings of this study are available from the corresponding authors on reasonable request.

## Results

### Study Population

Following the inclusion criteria, 18 LO-TLE and 24 MCI-AD were recruited from our previous study,^10^ together with 17 newly recruited HC, yielding a total of 59 participants. The LO-TLE cohort included 10 women and 8 men with a mean age of 64.3 years (±8.9, range 52–79) and a mean age at epilepsy onset of 62.4 years (±8.5, range 51–79). eTable 1 summarizes clinical and imaging details of the LO-TLE cohort. The median epilepsy duration was 1 year; 33% of patients (6/18) had less than 1 year; 39% (7/18) had 1–2 years; and 28% (5/18) had 4–8 years. Based on ictal semiology and long-term video-EEG monitoring, 72% (13/18) of LO-TLE were diagnosed with left TLE, 11% (2/18) with right TLE, and 16% (3/18) with bilateral TLE. Based on a seizure frequency classification^41^ adapted from our previous study,^10^ 33% (6/18) of patients experienced less than one seizure per year at enrollment, 33% (6/18) yearly seizures, 16% (3/18) monthly seizures, 11% (2/18) weekly seizures, and 5% (1/18) daily seizures. At the time of neuropsychological assessment, most patients (78%) were receiving antiseizure medications (ASMs); 50% (9/18) were on monotherapy, 22% (4/18) on dual therapy, and 5% (1/18) on polytherapy. Four patients (22%) had recently initiated ASM and were still undergoing titration at the time of neuropsychological evaluation (eTable 1). Response to ASMs was assessed during follow-up visits routinely scheduled at our department. The first visit was conducted 6 months after hospital discharge, and patients were subsequently evaluated on an annual basis with a mean follow-up of 3.53 years (±1.10). Drug-resistant epilepsy (DRE), defined according to ILAE criteria,^42^ was identified in 4 patients (22%). All MRI scans were reported as normal except for 2 cases with hippocampal sclerosis (11%). Based on visual inspection, the median Fazekas score was 1 (range 0–1). LO-TLE and MCI-AD did not differ in age, sex, education, age at onset, or disease duration. MCI-AD showed significantly lower MMSE scores than both LO-TLE and HC (Table 1).

**Table 1:** Demographic and Clinical Characteristics of Patients and Control Populations

	LO-TLE	MCI-AD	HC	Stat.	Sign.
N	18	24	17	—	—
Age (y)	64.33 ± 8.95	60.75 ± 8.09	60.88 ± 6.90	1.20^F^	0.30
Sex (M/F)	9/9	13/11	8/9	0.20^χ^	0.90
Education (y)	11.00 ± 3.25	11.50 ± 3.57	11.47 ± 2.80	0.13^F^	0.87
MMSE	28.67 ± 1.32	25.21 ± 3.41	29.41 ± 0.87	17.91^F^^a^	<0.001^a^
Age at onset (y)	62.39 ± 8.50	57.96 ± 7.76	—	0.55^t^	0.08
Disease duration (y)	1 (0–4)	2 (1–4)	—	139.50^U^	0.08

Abbreviations: AD = Alzheimer disease; HC = healthy control; LO-TLE = late onset temporal lobe epilepsy; MCI = mild cognitive impairment; MCI-AD = MCI due to AD; MMSE = Mini-Mental State Examination; t = independent sample *t* test; U = Mann-Whitney *U* test; χ = χ^2^ test; F = analysis of variance.

Data are presented as mean ± SD except for disease duration, which is reported as median (Q1–Q3). Age, education, age at disease onset, and disease duration are presented in years. MMSE is presented as raw scores.

a Significant results.

### Pattern of Cortical and Subcortical Atrophy

Consistent with the previous study,^10^ LO-TLE showed preserved cortical thickness, comparable with HC but clearly distinct from MCI-AD. By contrast, MCI-AD exhibited bilateral cortical atrophy, particularly in the temporal lobes and precuneus, along with reduced hippocampal and amygdalae volumes, more pronounced in the right hemisphere compared with both controls and LO-TLE. Unlike our previous report,^10^ no amygdala subnuclei atrophy was observed in the LO-TLE group. Results are summarized in eFigure 1 and in eTables 2 and 3.

### Cognitive Domains Performances

**Figure 1:**
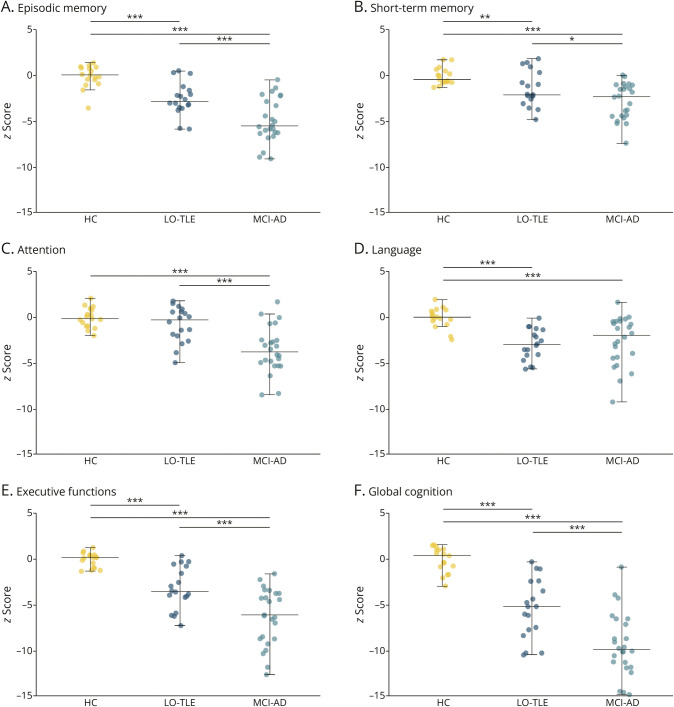
Cognitive Performance Across Domains in HC, LO-TLE, and MCI-AD Boxplots show the *z*-score distribution for healthy controls (HCs) on the left (yellow), late-onset temporal lobe epilepsy (LO-TLE) in the middle (blue), and mild cognitive impairment due to Alzheimer disease (MCI-AD) on the right (light blue) across the 6 cognitive domains investigated: (A) episodic memory, (B) short-term memory, (C) attention, (D) language, (E) executive functions, and (F) global cognition. Each dot represents a participant, the central horizontal lines represent the group means, while the upper and lower whiskers represent the maximum and minimum nonoutlier values within 1.5 × IQR of the distributions. The asterisks on the boxes indicate significant results from the group comparisons, specifically when ***p_FDR_ < 0.001, **p_FDR_ < 0.01, and *p_FDR_ < 0.05.

A limited proportion of missing data was observed across neuropsychological tests (4.7% ± 7.7%; range 0%–23.7%; eTable 4), with higher missingness for FCSRT measures. No participant had more than 20% missing data across the entire neuropsychological battery. The PC1 of the global cognition explained, on average across the 5 imputed data sets, 67% of the total variance. The corresponding loadings, which quantify the contribution of each neuropsychological test to the resulting global cognition score, are reported in eTable 5. Significant main effects of pathology emerged across all cognitive domains (Figure 1). In episodic memory (*F*_(2,53)_ = 29.50, *p* < 0.001), LO-TLE performed worse than HC (*t*_(53)_ = −7.79, *p*_FDR_ < 0.001) but better than MCI-AD (*t*_(53)_ = 3.55, *p*_FDR_ < 0.001). A similar pattern was found in short-term memory (*F*_(2,53)_ = 14.01, *p* < 0.001); LO-TLE obtained lower score than HC (*t*_(53)_ = −2.94, *p*_FDR_ = 0.007) but higher than MCI-AD (*t*_(53)_ = 2.10, *p*_FDR_ = 0.040). In attention (*F*_(2,53)_ = 16.44, *p* < 0.001), LO-TLE did not differ from HC yet outperformed MCI-AD (*t*_(53)_ = 3.97, *p*_FDR_ < 0.001). Language abilities (*F*_(2,53)_ = 10.77, *p* < 0.001) were reduced in LO-TLE compared with HC (*t*_(53)_ = 4.12, *p*_FDR_ < 0.001), with no significant differences between LO-TLE and MCI-AD. Finally, in executive functions (*F*_(2,53)_ = 32.10, *p* < 0.001), LO-TLE showed lower performance than HC (*t*_(53)_ = −3.76, *p*_FDR_ < 0.001) but higher performance than MCI-AD (*t*_(53)_ = 3.91, *p*_FDR_ < 0.001). Global cognition showed a robust effect of pathology (*F*_(2,53)_ = 45.26, *p* < 0.001), with LO-TLE performing worse than HC (*t*_(53)_ = −4.89, *p*_FDR_ < 0.001) and better than MCI-AD (*t*_(53)_ = 4.21, *p*_FDR_ < 0.001).

### Single-Test Performances

**Figure 2:**
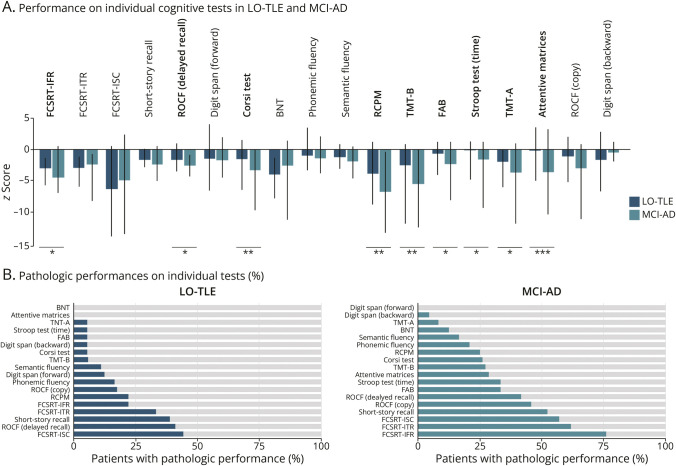
Performance on Individual Cognitive Tests in LO-TLE and MCI-AD (A) Bar plots display mean *z*-scores (±SDs) for late-onset temporal lobe epilepsy (LO-TLE, blue bars) and mild cognitive impairment due to Alzheimer disease (MCI-AD, light blue bars) across individual neuropsychological tests. The horizontal line at 0 indicates the mean cognitive performance of healthy controls (HCs) across each cognitive test. MCI-AD showed more pronounced impairments, particularly in episodic memory, executive functions, visuospatial memory, and language. Significant group differences are reported below each test as ***p_FDR_ < 0.001, **p_FDR_ < 0.01, and *p_FDR_ < 0.05. (B) Bar plots show the percentage of pathologic performances relative to the Italian normative cutoff values for each test in LO-TLE (left) and MCI-AD (right). LO-TLE showed higher impairment in episodic memory tasks, whereas MCI-AD exhibited widespread deficits across domains, with the highest rates in episodic memory, visuoconstructive abilities, and executive functions. BNT = Boston Naming Test; FAB = frontal assessment battery; FCSRT = Free and Cued Selective Reminding Test; IFR = Immediate Free Recall; ISC = Index of Sensitivity to Cueing; ITR = Immediate Total Recall; RCPM = Raven's colored progressive matrices; ROCF = Rey-Osterrieth Complex Figure; TMT-A = Trail Making Test Form A; TMT-B = Trail Making Test Form B.

**Figure 3:**
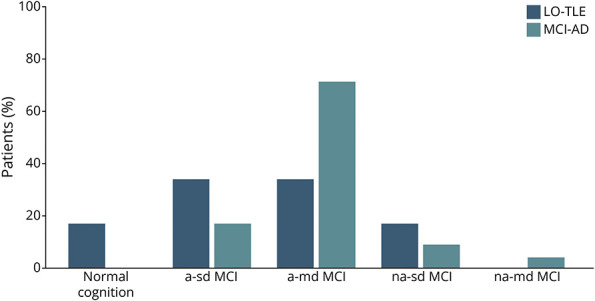
Distribution of Cognitive Phenotypes in LO-TLE and MCI-AD Bar plots show the distribution of mild cognitive impairment (MCI) phenotypes in late-onset temporal lobe epilepsy (LO-TLE, blue bars) and MCI due to Alzheimer disease (MCI-AD, light blue bars) expressed in percentage and classified based on the work of Petersen et al.^17^ as follows: normal cognition, amnesic single-domain MCI (a-sd MCI), amnesic multidomain MCI (a-md MCI), nonamnesic single-domain MCI (na-sd MCI), and nonamnesic multidomain MCI (na-md MCI).

Single-test results are presented in Figure 2 and in eTable 6. Overall, patients with LO-TLE performed better than the MCI-AD group, with the largest differences observed in episodic memory, reasoning, visual short-term memory, executive functions, and attention (Figure 2A). Binary analyses revealed group differences in pathologic performance rates according to Italian normative cutoffs (Figure 2B). In the LO-TLE group, pathologic performances were mainly confined to episodic memory measures. Impairment was most frequent on the FCSRT-ISC (44%), ROCF delayed recall (41%), and short-story recall (39%). FCSRT free and total recall were less affected (IFR: 22% and ITR: 33%). Other cognitive domains were largely preserved, with pathologic performances observed in most of the patients on language, attention, and executive function tests (≤20%, Figure 2B). By contrast, the MCI-AD group showed a broader and more severe cognitive impairment profile. Episodic memory was markedly affected, with pathologic performances on FCSRT-IFR (76%), FCSRT-ITR (62%), and FCSRT-ISC (57%), as well as on short-story recall (52%). Visuospatial memory was also frequently impaired, as indicated by pathologic ROCF delayed recall in 42% of patients. Executive and attentional deficits were common, with approximately one-third of patients showing pathologic performance on the Stroop test (33%), Trail Making Test–B (27%), and attentional matrices (29%) (Figure 2B). The MCI subtype distribution detected an MCI in 88% of LO-TLE and in all MCI-AD cases (Figure 3). Three patients with LO-TLE were classified as cognitively unimpaired (16%), 6 of them presented an a-sd MCI (33%), 6 an a-md MCI (33%), and 3 an na-sd MCI (16%), no one showed an na-md MCI. The majority of MCI-AD (17/24) presented an a-md MCI (70%), followed by 4 with an a-sd MCI (16%), 2 with an na-sd MCI (8%), and 1 with an na-md MCI (4%).

### CSF Biomarkers

**Figure 4:**
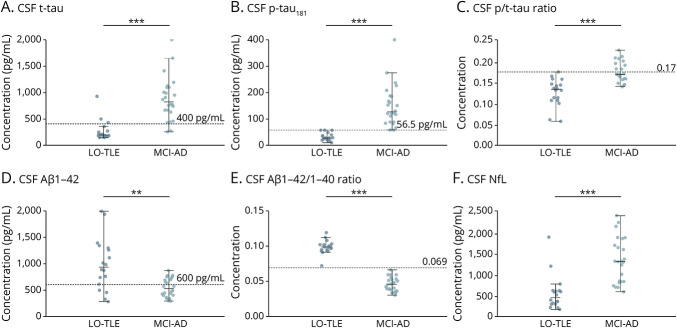
CSF Biomarkers in LO-TLE and MCI-AD Boxplots display CSF biomarker concentrations in late-onset temporal lobe epilepsy (LO-TLE) on the left (blue) and mild cognitive impairment due to Alzheimer disease (MCI-AD) on the right (light blue). Levels of total tau (t-tau), phosphorylated tau 181 (p-tau_181_), β-amyloid (Aβ) 1–42, and neurofilament light chain (NfL) are expressed in pg/mL, while Aβ_1–42/1–40_ and phosphorylated-to-total tau (p/t-tau) are expressed as ratios. Each dot represents an individual value; the central horizontal lines represent the group median, while the upper and lower edges of the box mark the 25th and 75th percentiles. The black dashed lines represent the cutoffs for each biomarker when available. The asterisks on the boxes indicate significant results from the group comparisons, specifically when ***p_FDR_ < 0.001 and **p_FDR_ < 0.01.

Patients with LO-TLE showed lower t-tau (*F*_(1,40)_ = 46.07, *p*_FDR_ < 0.001) and p-tau_181_ (*F*_(1,40)_ = 91.41, *p*_FDR_ < 0.001) levels compared with MCI-AD, together with higher Aβ_1–42_ (*F*_(1,40)_ = 11.5, *p*_FDR_ = 0.01) concentrations and Aβ_1–42/1–40_ ratio (*F*_(1,40)_ = 94.26, *p*_FDR_ < 0.001). According to the A/T framework, by design, all patients with LO-TLE resulted in A-/T- (eTable 1). CSF p/t-tau ratio levels differed significantly between patients, with LO-TLE showing lower p/t-tau concentrations compared with MCI-AD (*F*_(1,40)_ = 23.97, *p*_FDR_ < 0.001). All patients with LO-TLE fell below the previously proposed pathologic cutoff ^37^(i.e., <0.17). In 3 cases (subjects 7, 9, 96), the reduced ratio reflected elevated t-tau, whereas in the other patients, the value of the p/t-tau ratio seems to be driven by concomitant decrease of both p-tau_181_ and t-tau levels. Patients with LO-TLE additionally showed lower CSF NfL compared with MCI-AD (*F*_(1,40)_ = 39.26, *p*_FDR_ < 0.001). All results are summarized in Figure 4, and group-level descriptive statistics are reported in eTable 7.

### Correlations Analysis

**Figure 5:**
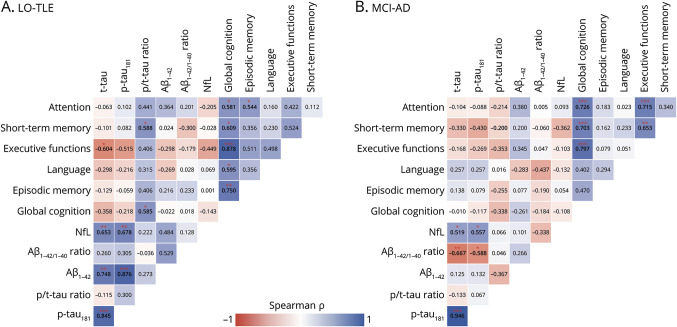
Correlations Between CSF Biomarkers and Cognitive Performances in LO-TLE and MCI-AD Heatmaps show Spearman correlation coefficients between CSF biomarkers (i.e., t-tau, p-tau_181_, Aβ_1–42_, Aβ_1–42/1–40_, and NfL) and performances in cognitive domains (i.e., global cognition, episodic memory, language, executive functions, short-term memory, and attention). The correlations for the late-onset temporal lobe epilepsy (LO-TLE) population are depicted in panel A, on the left. The correlations for the mild cognitive impairment due to Alzheimer disease (MCI-AD) population are shown in panel B, on the right. Blue colors indicate positive correlations, and red colors indicate negative correlations. Spearman correlation coefficients (*rs*) are reported within each box. Significance levels are expressed with red asterisks as ***p_FDR_ < 0.001, **p_FDR_ < 0.01, and *p_FDR_ < 0.05. Aβ = β-amyloid; NfL = neurofilament light chain; p-tau = phosphorylated tau; t-tau = total tau.

Figure 5 summarizes the biomarker-biomarker, cognition-cognition, and biomarker-cognition Spearman correlations in the LO-TLE and MCI-AD groups.

In the LO-TLE group, p-tau_181_, t-tau, and Aβ_1–42_ were strongly positively intercorrelated. Both p-tau_181_ and t-tau were also positively associated with NfL, suggesting a link between tau pathology and neuroaxonal damage. Global cognition was positively associated with all cognitive domains, along with a positive association between attention and memory. Regarding biomarker-cognition relationships, the p/t-tau ratio was positively correlated with global cognition (*rs* = 0.585, *p*_FDR_ = 0.032) and short-term memory (*rs* = 0.588, *p*_FDR_ = 0.032), indicating that lower values may be linked to poorer cognitive performances. In addition, t-tau was negatively correlated with executive function (*rs* = −0.603, *p*_FDR_ = 0.047), suggesting worse executive performance with higher t-tau levels (Figure 5A, eTable 8).

In the MCI-AD group, p-tau_181_ and t-tau were negatively correlated with the Aβ_1–42/1–40_ ratio, and positively associated with NfL. Global cognition correlated positively with attention, executive function, and short-term memory; attention was also positively associated with executive function, which in turn correlated positively with short-term memory. No significant biomarker-cognition correlations were observed in the MCI-AD group (Figure 5B, eTable 9).

## Discussion

This study provides new insights into LO-TLE knowledge, highlighting potentially distinctive cognitive and biological signatures that may help differentiate it from AD-related pathology.

Although our previous study^10^ demonstrated preserved cortical thickness and subcortical volumes in LO-TLE with normal CSF AD biomarkers, this work adds a neuropsychological dimension, revealing cognitive alterations that are not reflected by structural MRI measures. Despite most LO-TLE met criteria for an MCI diagnosis, their cognitive profile diverged from MCI-AD in both domain involvement and clinical severity. This dissociation is supported by biological findings, with patients with LO-TLE showing significantly lower NfL levels and p/t-tau ratios compared with MCI-AD. The p/t-tau ratio has been proposed as a diagnostic and prognostic biomarker across several neurodegenerative conditions characterized by marked neuronal loss, including traumatic brain injury,^43^ amyotrophic lateral sclerosis,^44^ parkinsonian syndromes,^45^ and frontotemporal dementia.^46^ In this context, the low p/t-tau ratio observed in our cohort raises the hypothesis that LO-TLE may reflect a distinct non-AD neurobiological process, potentially related to an alternative proteinopathy, consistent with the absence of a typical CSF AD profile.

Compared with HC, our patients with LO-TLE exhibited cognitive deficits, primarily affecting episodic memory, short-term memory, language, and executive functions, with preserved attention. Relative to MCI-AD, patients with LO-TLE performed better across most cognitive domains. Moreover, the clinical effect of cognitive dysfunction was considerably milder and more circumscribed in LO-TLE, involving specific domains rather than diffuse impairment. These findings partially align with previous evidence from LOUE populations. Older adults with TLE have been reported to show memory and language deficits resembling those observed in amnestic MCI (aMCI), although delayed recall was more severely affected in aMCI,^47^ in line with our results. Moreover, cognitive deficits have been described in up to 17% of individuals with LOUE, particularly in delayed verbal recall.^48^ In our LOUE cohort, episodic memory was the most affected domain, yet performance remained higher than in MCI-AD. Of interest, attention was preserved in LO-TLE, with performance comparable with HC, whereas MCI-AD showed clear deficits. This dissociation may be clinically relevant because a detailed assessment of attentional functioning might aid the differential diagnosis between LO-TLE and MCI-AD. Previous studies have used the Preclinical Alzheimer Cognitive Composite (PACC-5) to detect subtle Aβ-related cognitive decline.^48^ However, as some components of the PACC-5 (e.g., Digit Symbol) were not included in our neuropsychological battery, this composite score could not be computed in our cohort. Nevertheless, we derived an equivalent global cognitive index based on PCA-derived domain scores. This measure confirmed an intermediate cognitive profile in LO-TLE, worse than HC but consistently better than MCI-AD. Regarding MCI phenotypes, our findings differ from previous studies^9^ reporting a predominance of multidomain impairment in LOUE. In our cohort, instead, MCI subtypes in LO-TLE were more heterogeneously distributed, likely reflecting differences in the underlying biological phenotype. Notably, unlike earlier reports,^9^ in which partial Aβ alterations were identified in a subset of patients, our LO-TLE cohort showed no evidence of AD-type pathology. It is also important to note that previous LOUE studies^8,9,49^ included a heterogeneous phenotypic spectrum, encompassing focal (temporal and extratemporal) and generalized epilepsies. By contrast, this study examined a well-characterized and phenotypically homogeneous LO-TLE cohort. This approach emphasizes the value of studying homogeneous, phenotype-defined populations to disentangle the cognitive and biological heterogeneity within LOUE.

As a novel aspect of this study, we expanded the biological phenotyping of LO-TLE^6,7^ by exploring additional CSF biomarkers: p/t-tau ratio and NfL. The p/t-tau ratio has recently gained attention as a marker associated with unfavorable cognitive outcomes in patients with MCI and normal CSF AD biomarkers. Recently, a p/t-tau ratio below 0.17 has been proposed as a predictor of a twofold increased risk of conversion to dementia in Aβ-negative MCIs.^37^ Moreover, the p/t-tau ratio was associated with limbic atrophy and steeper cognitive decline. The authors suggested that this biomarker may help identify specific conditions driven by nonamyloid mechanisms, potentially including TDP-43–related processes such as frontotemporal lobe degeneration (FTLD) and limbic-predominant age-related TDP-43 encephalopathy.^37^ It has been shown that a low p/t-tau ratio discriminates FTLD-TDP from FTLD-tau, driven by reduced p-tau_181_ burden in TDP-43 proteinopathies.^46^ Here, our patients with LO-TLE exhibited significantly lower p/t-tau ratios than MCI-AD, with all values falling below the proposed cutoff of 0.17.^37^ This reduction was primarily driven by decreased p-tau_181_ and t-tau levels, although a small subset of patients showed elevated t-tau values. It is important that a higher p/t-tau ratio was associated with better global cognition and short-term memory, suggesting that reduced values may reflect increased vulnerability across different cognitive domains. Overall, these findings point to a tau dysregulation in our LO-TLE, possibly reflecting alternative mechanisms such as TDP-43-related or mixed proteinopathy, rather than the diffuse tau hyperphosphorylation and neuroaxonal injury characteristic of AD. Consistently, relatively low NfL levels suggest limited neuroaxonal damage, indicating that cognitive impairment in our LO-TLE may not be primarily driven by progressive neurodegeneration. Neuropathologic evidence further supports a potential link between TDP-43 pathology and epileptogenesis. A postmortem study on 114 individuals with pathologically confirmed AD reported a 12% incidence of epilepsy, markedly higher than in the general population.^50^ Importantly, AD cases with epilepsy exhibited a significantly greater burden of TDP-43 pathology in the medial temporal and limbic cortices compared with AD patients without epilepsy, while tau and α-synuclein loads did not differ significantly. The authors proposed that TDP-43 accumulation, especially when colocalizing with tau, may promote neuronal hyperexcitability and circuit instability in vulnerable temporal regions.^50^ These observations support the hypothesis that alternative, non-AD–related molecular mechanisms, potentially including TDP-43–related pathways, may contribute to epilepsy in the aging brain.

The main strength of this study lies in the inclusion of well-defined and homogeneous patient populations, evaluated within the same clinical setting using standardized procedures. The multimodal approach combining neuroimaging, neuropsychological assessment, and biomarker profiling allowed for a comprehensive characterization of cognitive and biological features in our LO-TLE cohort. However, our findings should be interpreted in the context of the studied population, which was restricted to patients with normal AD biomarkers, and may therefore not be generalizable to the broader LO-TLE population. Several limitations should be acknowledged. The relatively small and cross-sectional design precludes conclusions on cognitive trajectories over time. In addition, the absence of behavioral and psychiatric assessments limits a full understanding of the clinical profile. Notably, the presence of cognitive alterations in the absence of detectable morphometric differences between LO-TLE and HC suggests a dissociation between structural and functional measures, pointing to possible microstructural white matter changes not captured by conventional imaging. The sample size also precluded reliable comparisons between left-sided and right-sided LO-TLE. In this cohort, most patients had left-sided TLE, whereas right-sided or bilateral cases were underrepresented. Notably, a left-sided predominance has been similarly reported in other LOUE cohorts.^47,49^ It remains unclear whether this imbalance reflects a true biological predominance or the underrecognition of right-sided cases due to subtler clinical manifestations. Another limitation relates to the multiple statistical comparisons performed, particularly in the context of analyses based on PCA-derived cognitive measures. Although corrections for multiple testing were applied where appropriate, the number of repeated analyses may still increase the risk of type I error. Accordingly, these findings warrant confirmation in larger, independent, and ideally longitudinal cohorts using advanced imaging techniques sensitive to microstructural alterations, along with comprehensive behavioral assessments.

In conclusion, LO-TLE patients with normal CSF biomarkers showed a distinct cognitive profile compared with MCI-AD, particularly characterized by preserved attention in contrast to the marked attentional decline observed in MCI-AD. This dissociation may provide a useful clinical clue for differentiating these conditions in routine assessment, although it should be interpreted cautiously given the stringent selection criteria applied to our cohort, which may limit generalizability to the broader LO-TLE spectrum. Despite the absence of morphometric differences between LO-TLE and HC, cognitive deficits and a high prevalence of MCI were observed. Longitudinal multimodal studies are needed to determine whether LO-TLE represents an alternative neurodegenerative pathway or a relatively stable condition.

## Glossary

3D-FLAIR: 3-dimensional fluid-attenuated inversion recovery
Aβ: β-amyloid
AD: Alzheimer disease
aMCI: amnestic MCI
ANCOVA: analysis of covariance
a-md MCI: amnestic multiple-domain MCI
a-sd MCI: amnestic single-domain MCI
ASM: antiseizure medication
FCSRT: Free and Cued Selective Reminding Test
FDR: false discovery rate
FTLD: frontotemporal lobe degeneration
HC: healthy control
IFR: Immediate Free Recall
ILAE: International League Against Epilepsy
ISC: Index of Sensitivity to Cueing
ITR: Immediate Total Recall
LO-TLE: late-onset temporal lobe epilepsy
LOUE: late-onset unexplained epilepsy
MCI: mild cognitive impairment
MCI-AD: MCI due to AD
MMSE: Mini-Mental State Examination
na-md MCI: nonamnestic multiple-domain MCI
na-sd MCI: nonamnestic single-domain MCI
NfL: neurofilament light chain
PACC-5: Preclinical Alzheimer Cognitive Composite
PC1: first principal component
PCA: principal component analysis
p-tau: phosphorylated tau
p-tau181: phosphorylated tau 181
p/t-tau: phosphorylated-to-total tau ratio
ROCF: Rey-Osterrieth Complex Figure
t-tau: total tau
